# Data of de novo assembly and functional annotation of transcriptome of Peninsular Malaysian *Amomum* Roxb. species

**DOI:** 10.1016/j.dib.2023.109507

**Published:** 2023-08-21

**Authors:** Fathmath Shaman Fareed, Sam Yen Yen, Nallammai Singaram

**Affiliations:** a School of Biosciences, Faculty of Health and Medical Sciences, Taylor's University, Jalan Taylors, 47500, Subang Jaya, Selangor, Malaysia; b Forest Research Institute, Kepong, 52109, Kuala Lumpur, Selangor, Malaysia

**Keywords:** *Amomum*, Peninsular Malaysia, RNA, Transcriptome data, De novo assembly

## Abstract

The *Amomum* genus comprises of perennial, tropical, herbaceous plants that are distributed globally and possess both medicinal and ornamental properties. These plants contain a variety of secondary metabolites, including compounds with antioxidant and antimicrobial properties. This is the first transcriptomic analysis of seven *Amomum* species from Peninsular Malaysia, utilizing leaves, stems, and roots as sample material. Paired-end Illumina HiSeq technology was used for data generation which includes raw data, cleaned reads, de novo assembly, and functional annotation. The data is accessible via NCBI BioProject (PRJNA936673).

Specifications Table

**Table:** 

Subject	Plant Science
Specific subject area	Transcriptomics
Type of data	Table Figure
How the data were acquired	Illumina NovaSeq-6000 platform
Data format	Raw, analyzed
Description of data collection	Total RNA was extracted from young leaves, stems, and roots of seven *Amomum* species, that were collected during their initial flowering period. Individual tissue types of each plant species were subjected to RNA extraction and pooled to obtain total RNA. This total RNA was utilized for RNA sequencing. Biological homology and a consistent RNA extraction protocol Macherey & Nagel NucleoSpin® RNA Plant extraction kit) was utilized in this study.
Data source location	Taylor's University, Subang Jaya, Malaysia
Data accessibility	Repository name: NCBI Data identification number: PRJNA936673 Direct URL to data: https://www.ncbi.nlm.nih.gov/bioproject/PRJNA936673 The raw sequences have been deposited in the SRA database of NCBI (PRJNA936673). The co-assembled data file has been deposited in Mendeley Database. Link to data: https://data.mendeley.com/datasets/czfphhnf7p/1
Related research article	–

## 1 Value of the Data

• This is the first transcriptome data of seven Peninsular Malaysian *Amomum* species, which can be useful for the remaining species.
• The RNA-seq data can be utilized to predict genes and identify key genes involved in biosynthesis of secondary metabolites, such as flavonoids, steroids, and terpenoids.
• As *Amomum* species are important ornamental and medicinal plants, thus this data is significant for future genomic studies and gene expression analysis.
• These transcriptomic datasets can serve as reference for the design of targeted experiments to explore secondary metabolite biosynthesis pathways or investigate gene expression patterns in other *Amomum* species of Peninsular Malaysia, yet to be studied.

## 2 Objective

The objective of this study is to generate transcriptomic datasets using RNA-Seq technology to characterize and compare differences in important genes, pathways and networks that are involved in the production of secondary metabolites between selected *Amomum* species of Peninsular Malaysia.

## 3 Data Description

The following data is transcriptomic data of selected Peninsular Malaysian *Amomum* species using Illumina NoveSeq technology. Approximately 170 GB raw data was generated from total RNA of seven species (14 samples) including different plant tissues, namely, leaves, stem, and roots. The absorbance readings, RNA concentration, and RIN (RNA integrity number) values are provided in Supplementary Material, Table S1. Table 1 below shows the summary of raw and clean reads generated from the sequencing. A total of 806,223,725 clean reads were obtained for the 14 samples. The Q20 and Q30 percentages were > 96 and > 90 respectively. The GC% ranged from 48.12% to 51.86%. The de novo assembly produced 470,621,303 total nucleotides for transcripts with an average of 623 bp, and N50 of 674 and 177,787,746 nucleotides for Unigenes with average 592 bp and N50 of 342 (Table 2 and 3).

**Table 1:** Summary of RNA Seq- generated from seven *Amomum* species.

Sample	Raw reads	Clean reads	Error rate	Q20	Q30	GC %
*A. uliginosum S1*	63,477,605	62,825,062	0.03	97.88	93.87	49.97
*A. uliginosum S2*	54,206,276	53,472,309	0.03	97.98	94.11	50.14
*A. testaceum S1*	64,497,817	64,102,006	0.03	97.93	93.96	49.32
*A. testaceum S2*	55,758,675	55,145,304	0.03	97.28	92.24	49.6
*A. trilobum S1*	59,301,998	58,753,731	0.03	97.33	92.31	49.6
*A. trilobum S2*	51,598,493	51,163,716	0.03	97.31	92.28	50.25
*A. aculeatum S1*	59,905,159	58,685,546	0.03	97.39	92.38	46.54
*A. aculeatum S2*	51,980,823	51,403,682	0.03	97.8	93.62	48.12
*A. smithiae S1*	58,151,209	57,558,017	0.03	97.34	92.36	50.56
*A. smithiae S2*	57,829,579	57,221,929	0.03	96.41	90.5	48.95
*A. curtisii S1*	61,424,872	60,770,730	0.03	97.39	92.48	51.31
*A. curtisii S2*	61,373,151	60,932,285	0.03	97.44	92.57	50.98
*A. elan S1*	63,319,229	62,404,546	0.03	96.57	90.93	51.86
*A. elan S2*	52,317,756	51,784,862	0.03	97.28	92.23	51.78

**Table 2:** Assembly statistics of *Amomum* species of Peninsular Malaysia.

	300–500bp	500–1kbp	1–2kbp	>2kbp	Total
Number of transcripts	362,210	305,608	81,467	6064	755,349
Number of Unigenes	169,171	98,361	29,782	2875	300,189

**Table 3:** Length distribution of Transcripts and Unigenes.

	Min length	Mean length	Median length	Max length	N50	N90	Total nucleotide
Transcript	301	623	514	10,222	674	363	470,621,303
Unigene	301	592	461	10,222	635	342	177,787,746

The findings show that 14,346 unigenes, or 18.99% of the total transcripts, had coding sequences (CDS). A total of 1041 sequences, or 0.72% of them, had both start and stop codons. A total of 50,457 sequences (35.17%) were categorized as "5 prime partial len" and had only termination codon, while 622 sequences (0.433%) were classified as "3 prime partial len" and contained only initiation codon. A total of 32,805 sequences (22.86%) were classified as "internal len" because they lacked either an initiation or termination codon.

**Fig. 1:**
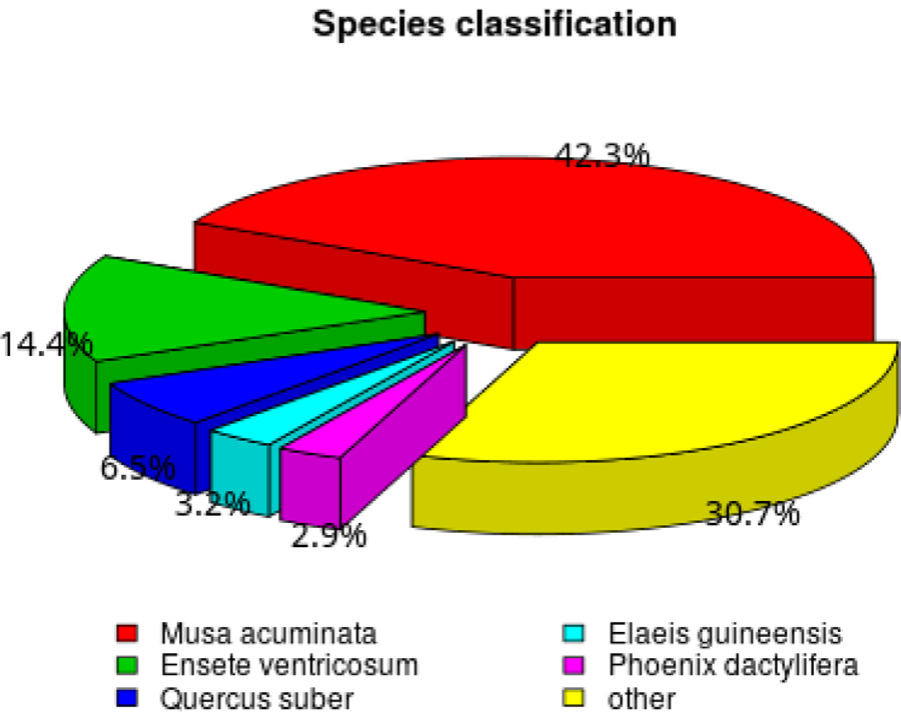
Species classification showing transcript information.

Of the 300,189 unigenes obtained, 158,064 were annotated unigenes in different databases. A total of 10,630 unigenes (3.54%) were annotated in all databases (NR, NT, KO, SwissProt, PFAM, GO, KOG). Among the annotated unigenes, 129,369 (43.09%) were obtained from NR, followed by 97,985 (32.64%) in SwissProt, 88,848 (29.59%) in PFAM, 88,836 (29.59%) in GO, 65,209 (21.72%) in NT, 55,925 (18.62%) and 30,966 (10.31%) in KO and KOG respectively (Fig. 1). More than 3760 of the mapped transcripts of *Amomum* share information from the five plant species, namely, *Musa acuminata, Ensete ventricosum, Quercus suber, Elaeis guineensis, Phoenix dactylifera* (Fig. 1).

**Fig. 2:**
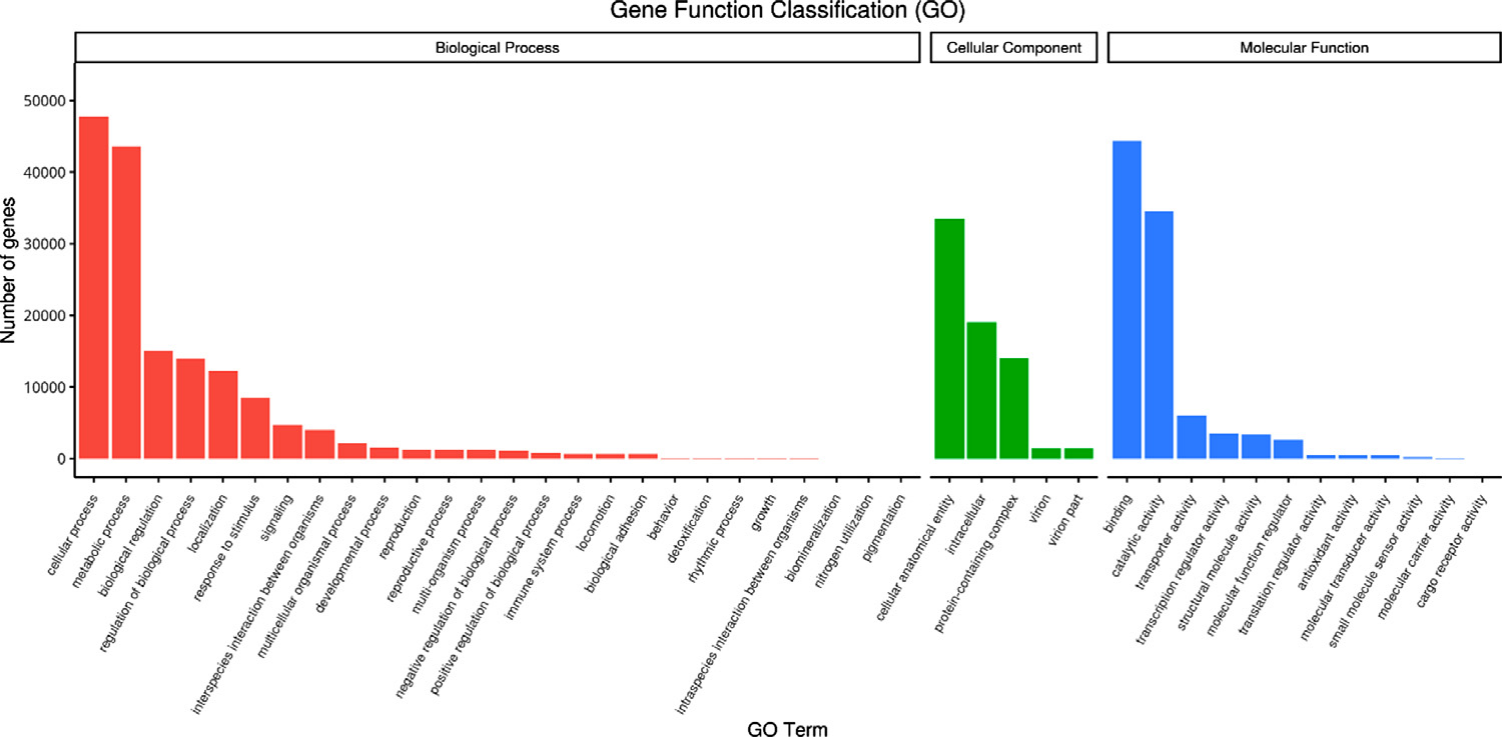
Summary of GO classification.

**Fig. 3:**
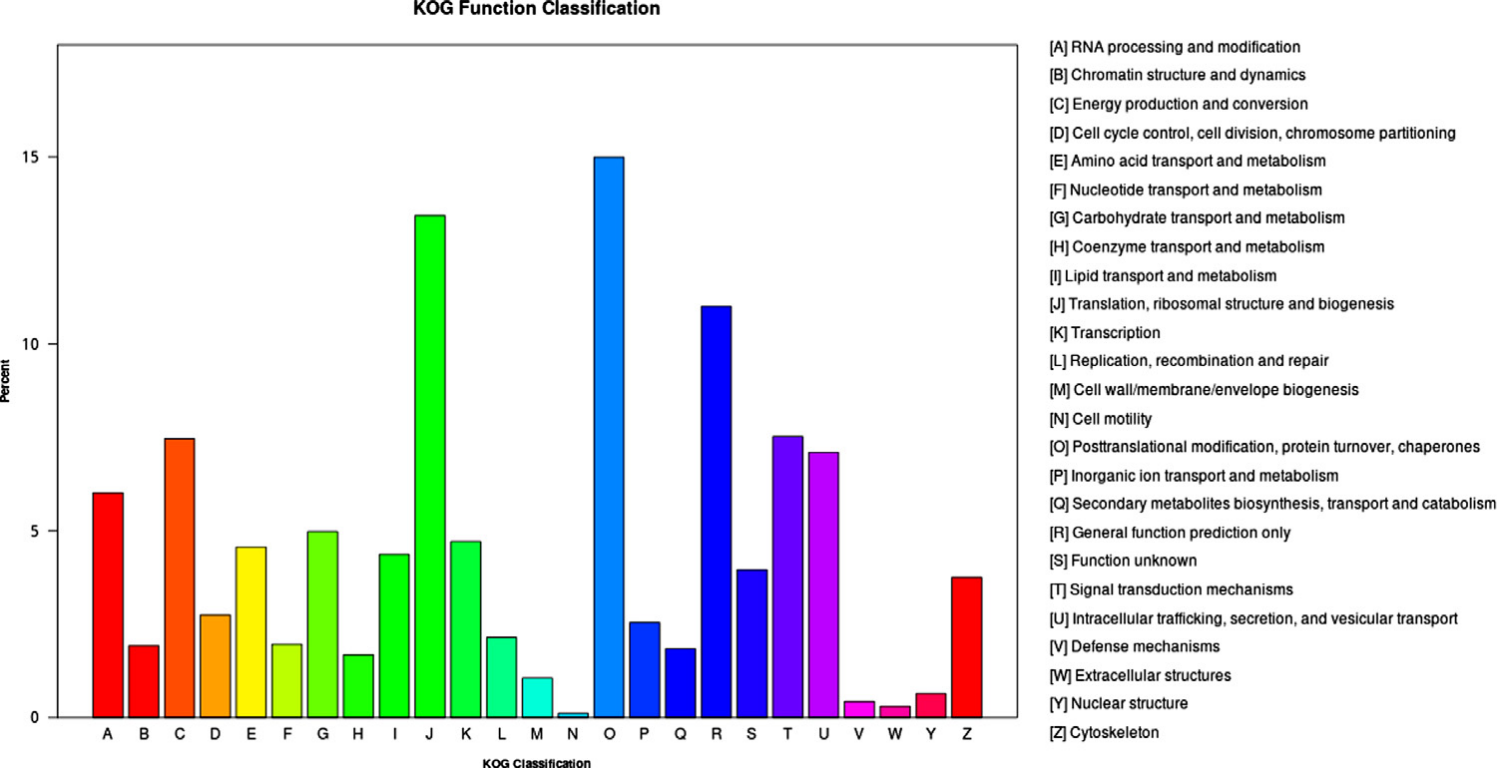
Summary of KOG classification.

**Fig. 4:**
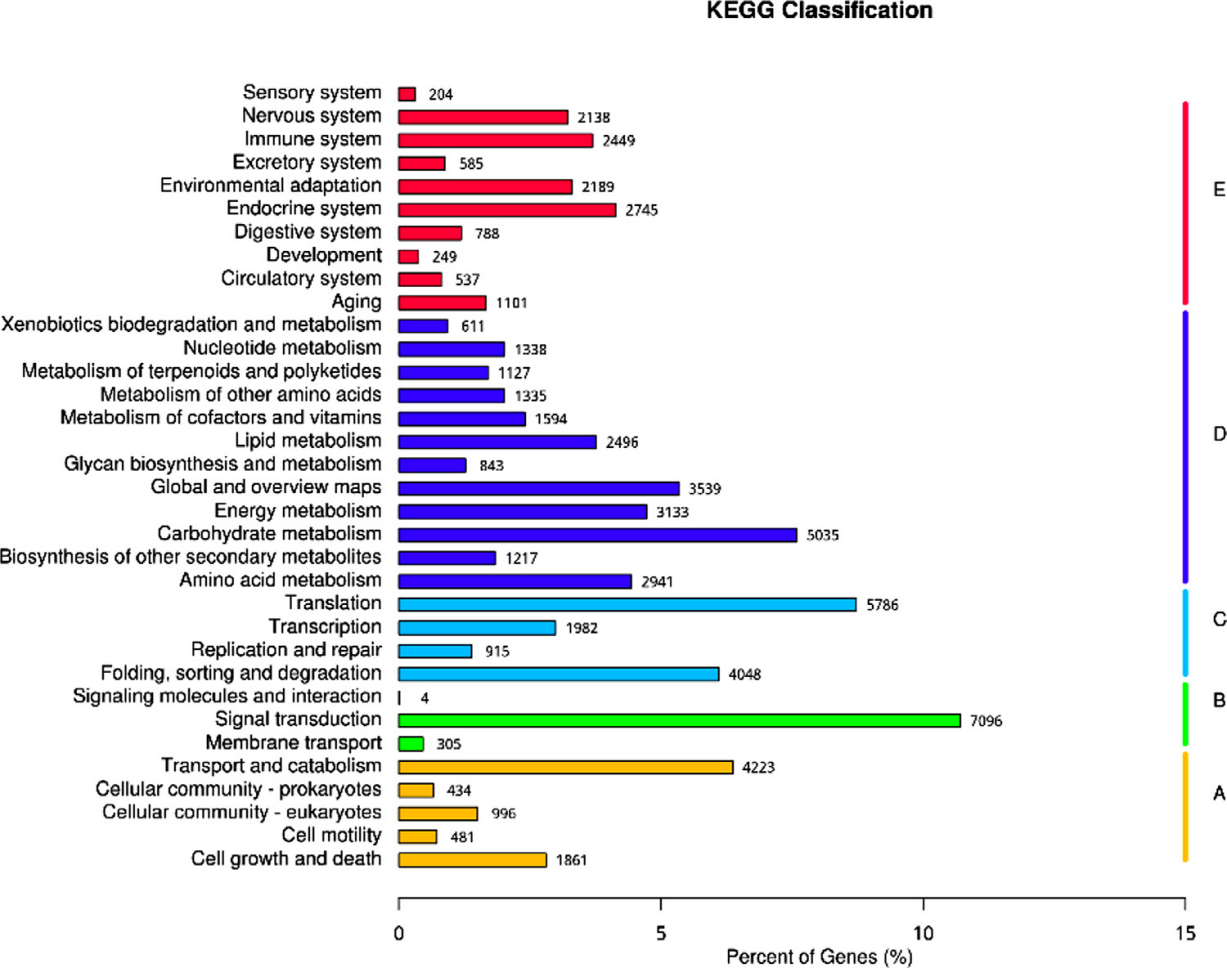
Summary of KEGG classification.

Gene Ontology (GO) classified all unigenes into three main GO domains: Biological Process, (BP), Cellular Component (CC), Molecular Function (MF). The highest number of genes (47,810) was for cellular process in biological process. The highest cellular component GO term consisted of 33,538 genes whereas binding (44,297) and catalytic activity (34,579) accounted for the highest in molecular function (Fig. 2). A total of 30,966 unigenes annotated in KOG and classified into 26 KOG functional categories (Fig. 3). The largest function category was Posttranslational modification, protein turnover, chaperones with 4643 unigenes followed by Translation, ribosomal structure, and biogenesis with 4159 unigenes. The maximum number of unigenes (7096) was assigned to Environmental Information Processing, followed by Genetic Information Processing (5786 unigenes). For metabolism of terpenoids and polyketides there were 1127 unigenes assigned and 1217 unigenes for biosynthesis of secondary metabolites (Fig. 4).

## 4 Experimental Design, Materials and Methods

### 4.1 Plant material

Three different young plant tissues, leaves, stem, and roots of seven *Amomum* species (*A. uliginosum, A. testaceum, A. elan, A. smithiae, A. trilobum, A. aculeatum,* and *A. curtisii*) were collected from their natural habitat, during their initial flowering period. The plant tissues were stored in RNALater solution upon collection to avoid any RNA degradation. Table 4 shows the list of *Amomum* species used in this study, with location and accession number.

**Table 4:** List of *Amomum* species with location and accession number.

Species	Accession Number	Location	GPS coordinates
*A. testaceum*	FRI97708	Batu Caves, Selangor	3°14′ N, 101°37′ E
*A. uligonosum*	FRI69087	Forest Research Institute of Malaysia, Selangor	3.14211° N, 101.413° E
*A. smithiae*	PBG 19005137	Penang Botanical Garden, Penang	5°26.42′ N, 100°17.32′ E
*A. aculeatum*	PBG 19005063	Penang Botanical Garden, Penang	5°26′ 30′' N, 100 °17′ 7′' E
*A. curtisii*	PBG 1900-5181	Penang Hill, Penang	5°25′ 09′' N, 100 °15′ 52′' E
*A. trilobum*	PBG 19001709	Penang Botanical Garden, Penang	5°26.50′ N, 100°17.29′ E
*A. elan*	PBG 19005014	Penang Botanical Garden, Penang	5°26′ 30′' N, 100 °17′ 7′' E

### 4.2 RNA extraction, cDNA library construction and RNA sequencing

RNA extraction was conducted using the optimized commercial kit protocol (Machery and Nagel RNA extraction kit) for each tissue type from each species, and the resulting RNA was pooled from all three plant tissue types to obtain total RNA. The RNA quality and quantity were assessed using a UV–visible spectrophotometer (Beckman Coulter, U.S.A). Each sample was measured for optical density (OD) at 230 nm, 260 nm, and 280 nm. RNA samples with RIN value of 6.5 or higher, purity readings of A_260/280_ ∼ 2.0 and A_260/230_ values ranging from 2.0 – 2.2 were sent to Apical Scientific, SDN, BHD, Malaysia (NGS service company) for preparation of cDNA libraries and subsequent transcriptomic sequencing (RNA-seq) using Next Generation Sequencing (NGS) technology (NovaSeq-PE150).

### 4.3 De novo assembly and functional annotation

The resulting sequence data was filtered to eliminate low quality reads using FASTQC [1]. The clean reads underwent de novo assembly using Trinity version 2.6.6 Program [2]. Corset version 4.6 [3] was employed to cluster transcripts, eliminate redundant sequences, and obtain unigenes. The protein coding sequences (CDS) were predicted using TransDecoder [4].

A series of bioinformatic analysis was performed using clean reads. The software RSEM v1.2.28 [5] was utilized for mapping and estimation of abundance of unigenes. Functional annotations were performed by BLASTx searches with e-value=1e-5 against NCBI non-redundance protein database (data downloaded in December 2019) as well as other established databases such as Swiss-Prot using Diamond version 0.8.22 (data downloaded in December 2022) [6], and Pfam using hmmscan HMMER 3.1 (data downloaded in November 2022) [7]. Blast2go (b2g4pipe_v2.5) [8] with parameters e-value = 1e-6 was used for assigning gene ontology (GO). Kyoto Encyclopedia of Genes and Genomes (KEGG) and Ortholog (KO) assignment and mapping of biosynthesis pathways were performed using KOBAS v3.0 [9] and GOSeq v1.32.0, topGO v2.32.0 respectively [10].

## Ethics Statement

Not applicable.

## CRediT Author Statement

**Fathmath Shaman Fareed:** Data curation, Writing – original draft; **Sam Yen Yen:** Supervision; **Nallammai Singaram:** Supervision, Conceptualization, Writing – review & editing.

## Data Availability

Data of de novo assembly and functional annotation of transcriptome of Seven Peninsular Malaysian Amomum Roxb. species (Original data) (Mendeley Data).

## Appendix Supplementary materials

Image, application 1

## References

[1] Bioinformatics B. (2011).

[2] Grabherr MG, Haas BJ, Yassour M, Levin JZ, Thompson DA, Amit I, Adiconis X, Fan L, Raychowdhury R, Zeng Q, Chen Z (2011). Trinity: reconstructing a full-length transcriptome without a genome from RNA-Seq data. Nat. Biotechnol..

[3] Davidson N.M., Oshlack A. (2014). Corset: enabling differential gene expression analysis for de novo assembled transcriptomes. Genome Biol..

[4] Haas B.J., Papanicolaou A., Yassour M., Grabherr M., Blood P.D., Bowden J., Couger M.B., Eccles D., Li B., Lieber M., MacManes M.D. (2013). De novo transcript sequence reconstruction from RNA-seq using the Trinity platform for reference generation and analysis. Nat. Protocols.

[5] Li B., Dewey C.N. (2011). RSEM: accurate transcript quantification from RNA-Seq data with or without a reference genome. BMC Bioinformatics.

[6] Buchfink B., Xie C., Huson D.H. (2015). Fast and sensitive protein alignment using DIAMOND. Nat. Methods.

[7] Potter S.C., Luciani A., Eddy S.R., Park Y., Lopez R., Finn R.D. (2018). HMMER web server: 2018 update. Nucl. Acids Res..

[8] Götz S., García-Gómez J.M., Terol J., Williams T.D., Nagaraj S.H., Nueda M.J., Robles M., Talón M., Dopazo J., Conesa A. (2008). High-throughput functional annotation and data mining with the Blast2GO suite. Nucl. Acids Res..

[9] Young M.D., Wakefield M.J., Smyth G.K., Oshlack A. (2010). Gene ontology analysis for RNA-seq: accounting for selection bias. Genome Biol..

[10] Alexa A., Rahnenführer J., Lengauer T. (2006). Improved scoring of functional groups from gene expression data by decorrelating GO graph structure. Bioinformatics.

