# Supplementary material for: Data of de novo assembly and functional annotation of transcriptome of Peninsular Malaysian Amomum Roxb. species
Source: Data Brief. 2023 Aug 21;50:109507. doi: 10.1016/j.dib.2023.109507 (PMC10493248; doi:10.1016/j.dib.2023.109507)
Supplement: Supplementary file 1 [file mmc1.docx]

Table S1: Nanodrop readings and RNA LabChip readings (Apical Scientific, Malaysia) of selected seven *Amomum* species

| Sample | Species | Source | Nanodrop Readings | | | LabChip Results | | | | | |
| --- | --- | --- | --- | --- | --- | --- | --- | --- | --- | --- | --- |
|  |  |  | **A_260/280_** | **A_260/230_** | **Con. ng/ul** | **A_260/280_** | **A_260/230_** | **Con. ng/ul** | **rRNA ratio** | **RIN** | **Total Amt (ug)** |
| A5-2 A. ulig leaf | *A. uliginosum* | leaf | 2.02 | 2.04 | 80.82 | 2.101 | 2.450 | 96.0 | 1.3 | 6.7 | 2.21 |
| A5-4 A. ulig stem | *A. uliginosum* | stem | 2.01 | 1.78 | 40.96 | 2.078 | 2.468 | 47.0 | 0.8 | 6.8 | 0.99 |
| A41-2 A. ulig roots | *A. uliginosum* | root | 2.1 | 1.48 | 50.63 | 2.162 | 1.934 | 57.0 | 0.8 | 6.4 | 1.60 |
| A42-2 A. ulig leaf | *A. uliginosum* | leaf | 2.05 | 1.81 | 232.7 | 2.203 | 2.510 | 132.0 | 0.9 | 6.4 | 3.70 |
| A41-4 A. ulig stem | *A. uliginosum* | stem | 2.1 | 2.19 | 315.77 | 2.171 | 2.309 | 176.0 | 1.3 | 6.9 | 4.93 |
| A44-3 A. ulig roots | *A. uliginosum* | root | 1.9 | 1.18 | 57.98 | 2.030 | 1.458 | 58.0 | 1.5 | 7.1 | 0.46 |
| S10 A. test leaf | *A. testaceum* | leaf | 2.04 | 2.08 | 107.3 | 2.115 | 2.446 | 128.0 | 1.2 | 6.6 | 2.30 |
| A10-3 A. test stem | *A. testaceum* | stem | 1.96 | 1.53 | 96.65 | 2.175 | 2.152 | 94.0 | 0.8 | 6.6 | 1.88 |
| A3-4 A. test roots | *A. testaceum* | root | 2.01 | 1.92 | 61.73 | 2.152 | 2.384 | 76.0 | 1.1 | 7.2 | 2.13 |
| A43-4 A. test leaf | *A. testaceum* | leaf | 1.92 | 1.71 | 48.51 | 2.061 | 2.344 | 57.0 | 1.1 | 6.8 | 1.60 |
| A10-4 A. test stem | *A. testaceum* | stem | 2.1 | 1.94 | 47.85 | 2.206 | 2.185 | 49.0 | 0.7 | 7.2 | 0.98 |
| A12-2 A. test roots | *A. testaceum* | root | 2.06 | 1.86 | 108.99 | 2.152 | 2.222 | 152.0 | 1.3 | 7.7 | 3.50 |
| A14-3 A. trilo leaf | *A. trilobum* | leaf | 1.97 | 1.68 | 58.31 | 2.110 | 2.306 | 52 | 0.6 | 6.4 | 1.30 |
| A50-2 A. trilo stem | *A. trilobum* | stem | 1.89 | 1.17 | 54.69 | 2.033 | 0.779 | 79.0 | 0.7 | 6.3 | 2.37 |
| A28-3 A. trilo roots | *A. trilobum* | root | 2.09 | 2.03 | 214.41 | 2.176 | 2.327 | 143.0 | 1.2 | 7.0 | 3.29 |
| A27-4 A. trilo leaf | *A. trilobum* | leaf | 2.09 | 2.25 | 271.54 | 2.187 | 2.424 | 173.0 | 1.1 | 6.3 | 3.98 |
| A48-1 A. trilo stem | *A. trilobum* | stem | 2.06 | 1.84 | 103.44 | 2.135 | 2.163 | 133.0 | 1.2 | 7.0 | 3.99 |
| A28-4 A. trilo roots | *A. trilobum* | root | 2.06 | 2.02 | 209.67 | 2.173 | 2.213 | 126.0 | 1.4 | 7.0 | 3.53 |
| A29-1 A. trilo leaf | *A. trilobum* | leaf | 1.95 | 1.47 | 65.7 | 2.090 | 1.784 | 90.0 | 0.9 | 6.9 | 2.52 |
| A37-3 A. acul stem | *A. aculeatum* | stem | 2.01 | 1.61 | 143.87 | 2.145 | 1.816 | 180.0 | 1.0 | 6.5 | 5.04 |
| A40-3 A. acul roots | *A. aculeatum* | root | 2.03 | 1.63 | 72.22 | 2.112 | 2.052 | 92.0 | 1.7 | 8.5 | 2.58 |
| A45-2 A. aculeleaf | *A. aculeatum* | leaf | 2.02 | 1.92 | 77.14 | 2.105 | 2.250 | 119.0 | 1.2 | 6.8 | 3.33 |
| A29-4 A. acul stem | *A. aculeatum* | stem | 2 | 1.61 | 83.02 | 2.109 | 1.996 | 110.0 | 1.1 | 7.1 | 3.08 |
| A30-2 A. acul roots | *A. aculeatum* | root | 2.02 | 1.71 | 46.68 | 2.136 | 2.161 | 51.0 | 1.4 | 8.0 | 1.43 |
| A48-3 A. smith leaf | *A. smithiae* | leaf | 1.98 | 1.25 | 47.09 | 2.123 | 1.357 | 77.0 | 1.0 | 6.8 | 2.31 |
| A45-3 A. smit stem | *A. smithiae* | stem | 2.08 | 1.51 | 54.69 | 2.031 | 1.569 | 59.0 | 0.6 | 6.5 | 1.65 |
| A36-3 A. smit roots | *A. smithiae* | root | 2.07 | 1.99 | 55.34 | 2.142 | 2.255 | 72.0 | 0.9 | 6.7 | 2.02 |
| A48-4 A. smith leaf | *A. smithiae* | leaf | 2.05 | 1.77 | 51.98 | 2.233 | 1.998 | 87.0 | 1.6 | 7.3 | 2.61 |
| A45-4 A. smith stem | *A. smithiae* | stem | 1.96 | 1.43 | 75.51 | 2.051 | 1.717 | 82 | 0.80 | 6.7 | 2.05 |
| A40-2 A. smith roots | *A. smithiae* | root | 2.07 | 1.87 | 115.14 | 2.173 | 2.160 | 133.0 | 1.3 | 7.4 | 3.72 |
| A30-3 A. curti leaf | *A. curtisii* | leaf | 2.09 | 2.19 | 279.86 | 2.139 | 2.453 | 163.0 | 0.9 | 6.6 | 4.56 |
| A31-1 A. curti stem | *A. curtisii* | stem | 2.07 | 1.82 | 76.5 | 2.085 | 2.280 | 91.0 | 1.0 | 6.9 | 2.55 |
| A41-1 A. curti roots | *A. curtisii* | root | 2.03 | 1.16 | 59.71 | 2.072 | 1.250 | 54.0 | 0.7 | 6.5 | 1.51 |
| A46-1 A. curti leaf | *A. curtisii* | leaf | 2.13 | 2.23 | 488.29 | 2.152 | 2.451 | 204.0 | 0.8 | 6.4 | 15.3 |
| A31-2 A. curti stem | *A. curtisii* | stem | 2.08 | 1.6 | 132.11 | 2.104 | 1.756 | 173.0 | 1.2 | 6.6 | 4.84 |
| A31-3 A. curti roots | *A. curtisii* | root | 2.11 | 1.86 | 37.25 | 2.160 | 2.306 | 48.0 | 0.6 | 6.4 | 1.34 |
| A49-4 A. elan leaf | *A. elan* | leaf | 2.09 | 2.15 | 215.77 | 2.183 | 2.315 | 177.0 | 1.4 | 6.8 | 10.62 |
| A38-1 A. elan stem | *A. elan* | stem | 1.98 | 1.07 | 37.17 | 2.110 | 1.394 | 35.0 | 0.8 | 7.2 | 0.81 |
| A13-3 A. elan roots | *A. elan* | root | 2.01 | 1.46 | 115.35 | 2.143 | 1.686 | 87.0 | 1.0 | 7.0 | 2.44 |
| A50-1 A. elan leaf | *A. elan* | leaf | 2.1 | 2.14 | 282.26 | 2.201 | 2.409 | 200.0 | 1.5 | 6.7 | 12.00 |
| A39-1 A. elan stem | *A. elan* | stem | 2.02 | 1.45 | 87.19 | 2.110 | 1.632 | 92.0 | 1.0 | 6.5 | 2.12 |
| A13-3 A. elan roots | *A. elan* | root | 2.01 | 1.46 | 115.35 | 2.143 | 1.686 | 87.0 | 1.0 | 7.0 | 2.44 |
